# Optimizing inter-joint distances of robotic forceps for vertical needle driving in pediatric surgery: a virtual reality simulator study

**DOI:** 10.1007/s11548-025-03535-6

**Published:** 2025-10-29

**Authors:** Kota Aono, Kazuya Kawamura, Daisuke Akimitsu, Michito Katayama, Reiko Takahashi, Hikaru Terazawa, Masakazu Murakami, Satoshi Ieiri

**Affiliations:** 1 Graduate School of Science and Engineering, Chiba University, Chiba, Japan; 2 Center for Frontier Medical Engineering, Chiba University, Chiba, Japan; 3 Department of Pediatric Surgery, Kagoshima University, Kagoshima, Japan

**Keywords:** Robotic surgery, Robotic forceps, Pediatric surgery, Virtual reality, Congenital biliary dilatation in children

## Abstract

**Purpose:**

While related studies have explored robotic forceps adaptations for narrow surgical workspaces, most have focused on horizontal needle driving, with limited research on optimizing robotic forceps configurations for vertical needle driving in pediatric choledochojejunostomy. Moreover, the impact of inter-joint distance adjustments on motion volume and obstructed value for vertical needle driving remains unclear, necessitating further investigation. We aimed to evaluate the effect of inter-joint distances in robotic forceps on a needle driving task that simulated vertical needle driving in a choledochojejunostomy for congenital biliary dilatation in children using a virtual reality simulator.

**Method:**

We examined the relationship between variations in inter-joint distances, motion volume, and obstructed value. Four pediatric surgeons performed an experimental task, passing a needle through two rings using the right robotic forceps. Based on these results, the inter-joint distances were adjusted through an optimum design approach, which adopted the weighted $$l_{p} $$ norm method. We then compared the robotic forceps before and after optimization to evaluate changes in the motion volume and obstructed value.

**Results:**

We observed a trade-off between motion volume and obstructed value in vertical needle driving. Adjusting inter-joint distances improved motion volume for Participants A, B, and C. However, obstructed value did not improve across all participants. This was attributed to the five-joint robotic forceps used in the study. The impact of inter-joint distances on the obstructed value may be limited when the number of joints remains constant.

**Conclusion:**

We verified the impact of inter-joint distances on vertical needle driving, considering the narrow surgical workspace and the specific requirements of pediatric surgery. Our findings suggest that adjusting inter-joint distances can improve motion volume in vertical needle driving. However, further investigation is needed to assess its effects across different joint configurations.

## Introduction

### Background

Endoscopic surgery for congenital biliary dilatation (CBD) in children requires advanced techniques to navigate the narrow surgical workspace due to the small size of the patient [1–3]. During a choledochojejunostomy for CBD, the suture needle must be passed from the bile duct to the jejunum in the depth direction (vertical needle driving) within the endoscopic view. The use of surgical robots has been considered to address these challenges [1, 4, 5]. Compared to humans performing conventional endoscopic surgery, surgical robots enhance operating accuracy through features such as motion suppression and motion scaling [6]. Additionally, the multi-joint structure of robotic forceps (RF) imposes fewer motion constraints, facilitating suturing and ligation in narrow surgical workspaces more effectively than endoscopic forceps [5, 7, 8]. However, robot arms and RF designed for adult patients are too large for newborns and infants, as their surgical workspace is significantly smaller [5, 7, 9]. Hence, although surgical robots hold promise for improving accuracy in pediatric surgery, their application to newborns and infants remains challenging. Developing surgical robots suitable for pediatric surgery requires designing structures that can operate efficiently in confined workspaces while supporting complex techniques specific to pediatric procedures [10, 11].

### Related works

To adapt to narrow surgical workspaces, several studies have explored methods to reduce the diameter of RF. Intuitive Surgical Inc. reduced the RF diameter of the da Vinci® system (Intuitive Surgical Inc., CA, USA) from 8 to 5 mm. Although this reduction was achieved by increasing the tip length, the tip exhibited greater movement during flexion than before, rendering its use in confined surgical workspaces challenging [12, 13]. Marinho et al. reduced the RF diameter to 3.5 mm and demonstrated the feasibility of performing suturing operations on a newborn chest model [14]. Kato et al. performed robotic surgery on three pediatric patients using 3 mm and 5 mm forceps attached to the Senhance® Surgical System (Senhance®) (Asensus Surgical, NC, USA). Their results confirmed that thinner forceps enable accurate and safe procedures in pediatric surgery. However, the absence of a joint at the tip of the Senhance® RF was reported to limit its range of motion compared to the da Vinci® system [15].

Adapting RFs to narrow surgical workspaces requires consideration not only of their diameter but also of their tip joint structure. Kayser et al. performed horizontal suture driving (suturing in the width direction of the endoscopic view) in simulated narrow surgical workspaces using the Versius® Robotic System (Versius®) (Cambridge Medical Robotics Ltd., Cambridge, UK). The feasibility and safety of horizontal suture driving using Versius® in a narrow surgical workspace were confirmed. Additionally, the structure of this RF was compared with those of da Vinci® and Senhance® by analyzing the results of this study alongside those of related studies [12, 16–18]. The shorter tip length of this RF, compared to the 5 mm RF of da Vinci®, resulted in reduced movement, rendering operation easier. The multi-joint structure of this RF facilitates complex reconstruction procedures in confined surgical spaces more effectively than the RF of Senhance®, which has fewer joints [19]. Bandara et al. developed an RF designed for narrow surgical workspaces by evaluating the performance of a 3.5 mm diameter RF with four degrees of freedom (DOF) and an elastic mechanism [20]. Kawamura et al. investigated the optimal design of RF tip parameters for horizontal needle driving using a virtual reality simulator (VRS) to replicate the surgical workspace in pediatric surgery. They assessed the RF’s motion volume, representing the extent of movement within the body cavity, and the obstructed value, which indicates the percentage of the endoscopic field blocked by the RF. Their findings revealed a trade-off between motion volume and obstruction, leading to the optimization of RF tip parameters based on these metrics [21]. These results suggest that optimizing RF tip design enhances its suitability for pediatric surgery. Furthermore, other design parameters may influence the optimal tip parameters of RF [22].

Although several studies have explored RF tip joint structures for use in narrow surgical workspaces, limited research has focused on RF tip structures specifically optimized for pediatric surgery in vertical needle driving.

### Purpose

As mentioned earlier, although research on adaptations for narrow surgical workspaces has been conducted, little attention has been given to difficult techniques such as vertical needle driving. In this study, we focused on the tip structure of the RF, which supports vertical needle driving and is essential for choledochojejunostomy in CBD. Aono et al. investigated changes in the obstructed value during a vertical needle driving task using RFs with varying numbers of joints at the tip, placed within the body cavity using a VRS [23]. In this experiment, the joint structure excluding the joint for opening and closing of the jaw was set to three, four, and five joints, and the results for each were compared. Their findings indicated that the five-joint RFs tended to have the lowest obstructed value. Similarly, Akimitsu et al. examined changes in motion volume during a vertical needle driving task using RFs with different numbers of joints at the tip, also evaluated with VRS [24]. In this experiment, the joint structure was also set to three, four, and five joints, and the results for each were compared. Their results showed that the five-joint RFs exhibited the largest motion volume. These findings highlight the necessity of carefully considering the number of joints required for optimal adaptation to a narrow surgical workspace. As a preliminary study, we examined the relationship between the length of the bending section (wrist length) of the five-joint RF, the motion volume, and the obstructed value. Our results confirmed a trade-off between motion volume and obstructed value. Additionally, we observed that variations in inter-joint distances could influence motion volume and obstructed value. This was attributed to changes in the radius of curvature at the RF tip during vertical needle driving, which resulted from modifications in wrist length. Therefore, in this study, we selected an RF with a 5-joint structure as the target joint structure and applied an optimum design method from a previous study [21] to adjust the inter-joint distances and aimed to evaluate their effect on a needle driving task that simulated vertical needle driving in choledochojejunostomy for CBD using VRS.

## Experiment

All participants were provided with information about the study, including its purpose, procedures, potential risks, and benefits before participation. Written informed consent was obtained from all participants. The participants were four pediatric surgeons (42.5 ± 5.0 [years]). Personal experiences and operating tendencies of pediatric surgeons may vary. This suggests that the effect of the tip joint structure of the RF on the motion volume and obstructed value may differ for each pediatric surgeon. Therefore, each participant was evaluated individually.

### Experimental environment

**Fig. 1:**
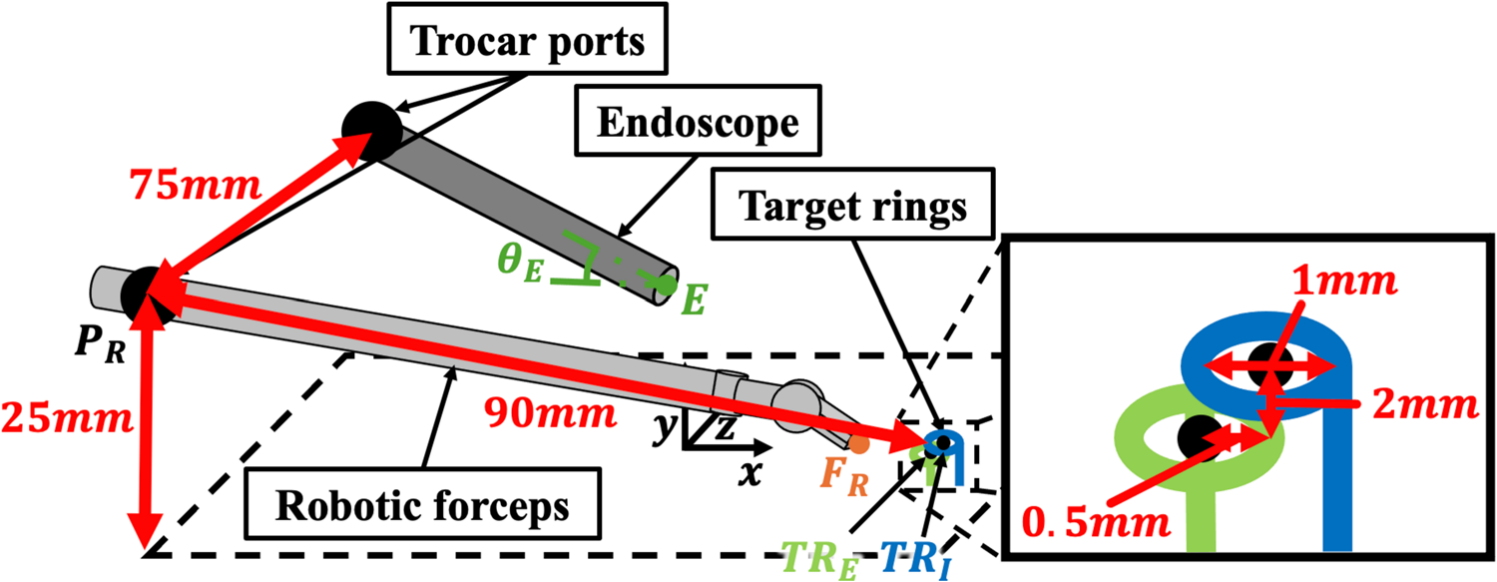
Experimental environment

In consultation with pediatric surgeons, an environment simulating choledochojejunostomy for the CBD was established in the VRS. Figure 1 and Table 1 show the experimental conditions, such as the positions of the two target rings for simulating the needle driving task, trocar port, and endoscope position. The endoscope was inserted at a 10° angle to the plane and fixed in position, assuming the use of a 30° telescope in choledochojejunostomy for the CBD, as advised by pediatric surgeons. The distance of the ring height was set to 2 mm, considering the thickness of the bile duct. Touch™ (3DSystems, SC, USA) was used as the controller.

**Table 1:** Initial parameters of the experimental environment

Data items		Parameters		Data items		Parameters	
Position of the trocar	*P*_*R*_	*x*	− 86.5 mm	Position of the insertion ring	*TR*_*I*_	*x*	5.5 mm
		*y*	25.0 mm			*y*	3.0 mm
		*z*	− 75.0 mm			*z*	0.0 mm
Initial position of the right robotic forceps	*F*_*R*_	*x*	0.5 mm	Position of the extraction ring	*TR*_*E*_	*x*	5.0 mm
		*y*	5.0 mm			*y*	1.0 mm
		*z*	− 5.0 mm			*z*	0.0 mm
Position of the endoscope	*E*	*x*	− 10 mm	Angle of the endoscope with the horizontal	*Θ*	40.0 °C	
		*y*	18.5 mm				
		*z*	0 mm				

### Experimental task

**Fig. 2:**
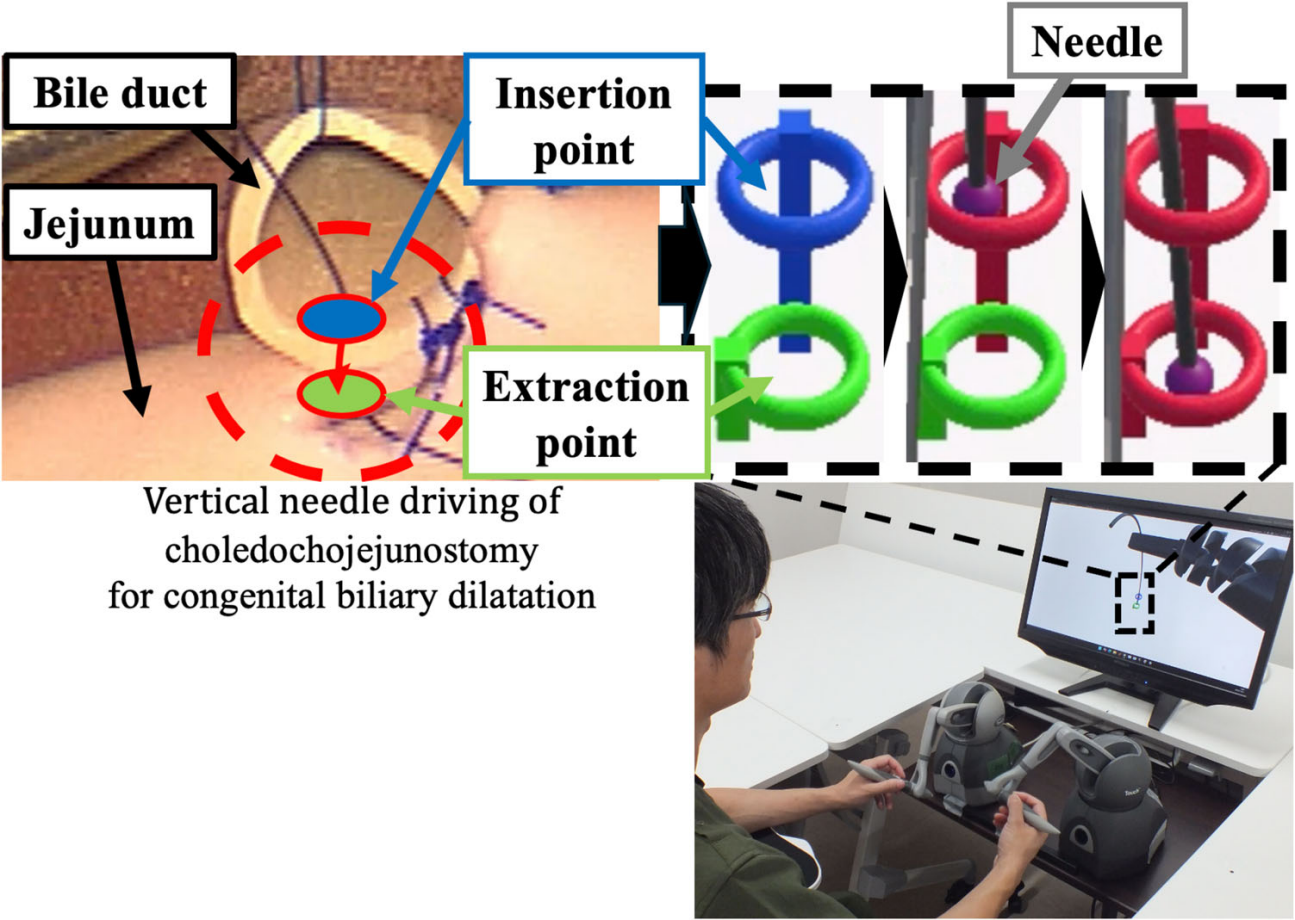
Experimental task

Although the vertical needle driving task required the coordinated operation of both the right and left RF, the functional requirements for each RF differed. In this study, we focused on the structure of the right RF to examine the effect of variations in inter-joint distances on vertical needle driving. The left RF assisted the right RF by applying tensile force to the target organ during the needle driving task. Given that the left RF maintained tension, the insertion and extraction points of the needle could be considered fixed. Based on this assumption, the vertical needle driving operation in choledochojejunostomy for the CBD comprises six steps: (1) vertical insertion at the bile duct insertion point, (2) vertical needle driving from the insertion point to the extraction point in the bile duct, (3) vertical extraction at the bile duct extraction point, (4) vertical insertion at the jejunum insertion point, (5) vertical needle driving from the jejunum insertion point to the extraction point, and (6) vertical extraction at the jejunum extraction point. For the experimental task, we simulated Steps (1) and (2) by having the right RF pass a needle through two rings. Step (1) was represented by passing the needle through a blue ring, while Step (2) was simulated by passing the needle through a yellow-green ring after passing through the blue ring (Fig. 2).

### Collected data

In this experiment, we measured the motion volume and obstructed value within the body cavity during the experimental task. Meehan reported that a lack of visual information in the endoscopic view affects treatment outcomes in the narrow surgical workspace of pediatric surgery [25]. Considering these aspects, we deemed it essential to evaluate the adaptability of the RF to narrow surgical workspaces by assessing not only its movement within the workspace but also the extent to which it obstructs the endoscopic view. Consequently, motion volume and the obstructed value were selected as key evaluation metrics. All data were collected from the initial position, which corresponded to the experiment’s starting position, until its completion.

**Fig. 3:**
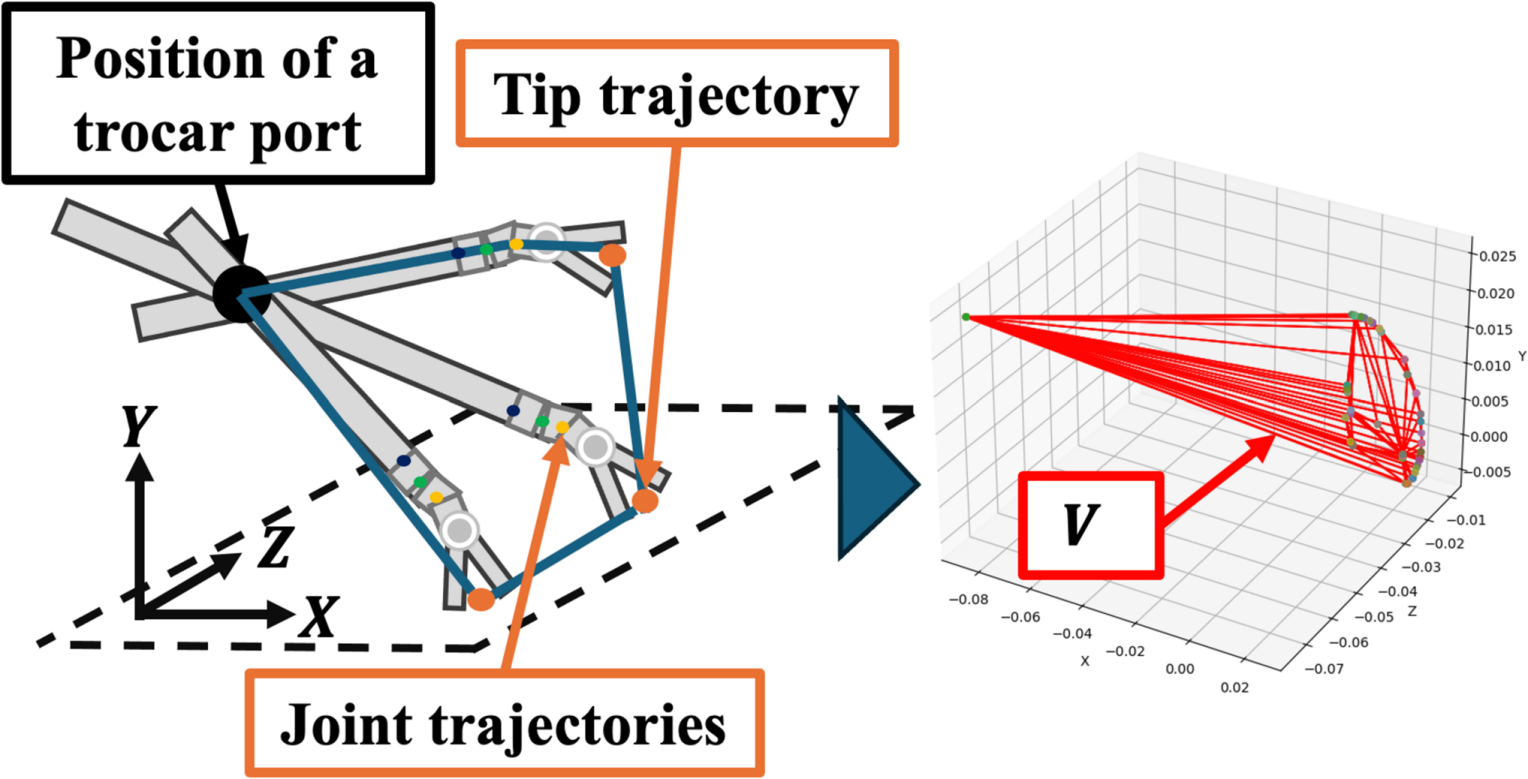
Motion volume

Motion volume was defined as the spatial volume occupied by the RF within the body cavity during the experimental task. To calculate this, we plotted the coordinates of the insertion port, RF joints, and tip trajectory in three-dimensional space. Using convex methods, we derived the smallest polyhedron encompassing all these coordinates; subsequently, the volume of the polyhedron was computed (Fig. 3). The polyhedron’s derivation via the convex method and the volume calculation were performed using Eq. (1), where $V$ represents the motion volume and $f\left( {x,y,z} \right)$ defines the polyhedron. These computations were carried out using the SciPy library in Python.

1 $$ V = \iiint_{V} {f\left( {x,y,z} \right){\text{d}}V} $$

**Fig. 4:**
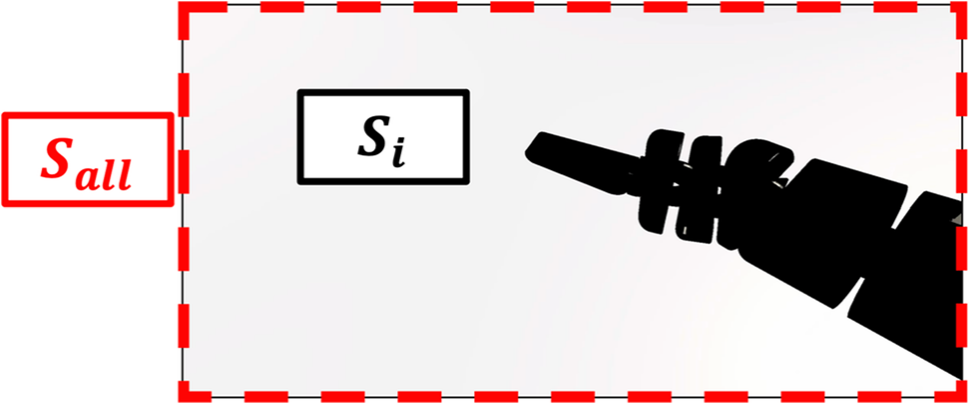
Obstructed value

The obstructed value was defined as the ratio of the number of pixels occupied by the RF to the total number of pixels in the endoscopic view during the experiment. The VRS view was recorded at 60 fps from start to end. The recorded footage was processed by masking. First, the image of the RF was extracted and binarized to count the number of pixels. The recorded footage was converted from RGB to HSV images, and masking was performed by specifying the color range of the RF using OpenCV (OpenCV.org, CA, USA). The obstructed value was then calculated using Eq. (2), where $$S$$ represents the obstructed value, $$n$$ is the number of frames, $$i$$ is the frame number, $$ S_{i}$$ is the number of pixels in the background of frame $$i$$, and $$ S_{{{\text{all}}}}$$ is the number of pixels in the endoscopic view (Fig. 4).

2 $$ S = \frac{1}{n}\mathop \sum \limits_{i = 1}^{n} \left( {1 - \frac{{S_{i} }}{{S_{{{\text{all}}}} }}} \right) $$

### Experimental method

(A) Verification of the relationship between inter-joint distances and the motion volume and obstructed value

**Fig. 5:**
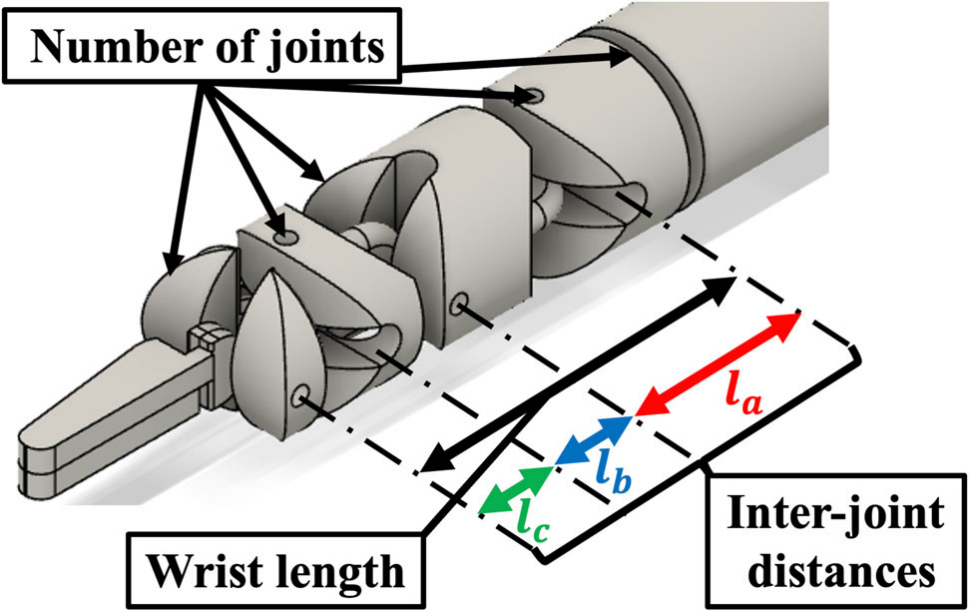
Parameters of the tip of the robotic forceps

We measured the change in the motion volume and obstructed value according to the difference in the inter-joint distances using the VRS. The experimental conditions were set to the inter-joint distances of the right five-joint RF, which had the smallest obstructed value in a previous study [23]. If the inter-joint distances are extremely long, they may not fit into a narrow surgical workspace. However, if the inter-joint distances are extremely short, the range of motion of the joint may be reduced owing to collisions between the links of the wrist during flexion. Therefore, when the inter-joint distances were set as $l_{a}$, $l_{b}$, and $l_{c}$ mm, as shown in Fig. 5, the side constraints for $l_{a}$, $l_{b}$, and $l_{c}$ were set as Eqs. (3)–(5), and the 27 types of RF listed in Table 2 were verified. The RF diameter was set to 5.0 mm.

3 $$ 2.0 \le l_{a} \le 5.0 $$

4 $$ 2.0 \le l_{b} \le 5.0 $$

5 $$ 2.0 \le l_{c} \le 5.0 $$

**Table 2:** Parameters of robotic forceps (i)–(xxvii)

No	Parameters [mm]			No	Parameters [mm]			No	Parameters [mm]		
	*l*_*a*_	*l*_*b*_	*l*_*c*_		*l*_*a*_	*l*_*b*_	*l*_*c*_		*l*_*a*_	*l*_*b*_	*l*_*c*_
(i)	2.0	2.0	2.0	(x)	3.5	2.0	2.0	(xix)	5.0	2.0	2.0
(ii)	2.0	2.0	3.5	(xi)	3.5	2.0	3.5	(xx)	5.0	2.0	3.5
(iii)	2.0	2.0	5.0	(xii)	3.5	2.0	5.0	(xxi)	5.0	2.0	5.0
(iv)	2.0	3.5	2.0	(xiii)	3.5	3.5	2.0	(xxii)	5.0	3.5	2.0
(v)	2.0	3.5	3.5	(xiv)	3.5	3.5	3.5	(xxiii)	5.0	3.5	3.5
(vi)	2.0	3.5	5.0	(xv)	3.5	3.5	5.0	(xxix)	5.0	3.5	5.0
(vii)	2.0	5.0	2.0	(xvi)	3.5	5.0	2.0	(xxv)	5.0	5.0	2.0
(viii)	2.0	5.0	3.5	(xvii)	3.5	5.0	3.5	(xxvi)	5.0	5.0	3.5
(ix)	2.0	5.0	5.0	(xviii)	3.5	5.0	5.0	(xxvii)	5.0	5.0	5.0

In this experiment, some variations may occur owing to the operation of each participant. This could cause ambiguity in verifying the factors that caused the change in the measured results when performing experiments that included these variations. To eliminate variations, we measured the trajectory of the moving needle using one type of RF for each participant and applied the trajectory data to all experimental conditions. First, each participant performed the experimental task five times using RF (i) after becoming familiar with it. Next, we obtained the input data by averaging the tip positions and postures measured during the task. Subsequently, we conducted an automatic simulation using their data with RF (i)–(xxvii) and measured the motion volume and obstructed value of RF (i)–(xxvii) for each participant.

(B) Optimum design based on the measured motion volume and obstructed value

We calculated the relationship formula using the measured results and adjusted the inter-joint distances using an optimum design approach. The inter-joint distances of the five-joint RF, denoted as $$l_{a}$$, $$l_{b}$$, and $$l_{c}$$, were selected as design variables, whereas the motion volume and obstructed value served as objective functions. The side constraints for $$l_{a}$$, $$l_{b}$$ and $$l_{c}$$ were set using Eqs. (3)–(5). The results for each participant presented in Sect. "Results"(A) were normalized using Eq. (6), where $$Y$$’ represents the normalized data, $$Y$$ is the original data, $$Y_{{{\text{max}}}}$$ is the maximum value of the original data, and $$Y_{{{\text{min}}}}$$ is the minimum value of the original data. Approximate equations for the design variables and the normalized objective functions were obtained using multiple regression analysis. The Pareto frontier was then determined using the weighted $$l_{p} $$ norm method, a multi-objective optimization technique. The weighted $$l_{p}$$ norm method is defined by Eq. (7), where $$p$$ is set according to the shape of the Pareto frontier. The relationship between the weight of the motion volume and the weight of the obstructed value is given by Eq. (7), where $$w_{{\text{V}}}$$ represents the weight of the motion volume and $$w_{{\text{S}}}$$ represents the weight of the obstructed value. In this study, $$p$$ was set to the value specified in Eq. (9), based on a previous study [21].

6 $$ Y^{\prime} = \frac{{Y - Y_{{{\text{min}}}} }}{{Y_{{{\text{max}}}} - Y_{{{\text{min}}}} }} $$

7 $$
 {\text{min}}\left| {\left| {F\left( {l_{a} ,l_{b} ,l_{c} } \right)} \right|} \right| = {\text{min}}\left[ {\left\{ {w_{{\text{V}}} V\left( {l_{a} ,l_{b} ,l_{c} } \right)} \right\}^{p} + \left\{ {w_{{\text{S}}} S\left( {l_{a} ,l_{b} ,l_{c} } \right)} \right\}^{p} } \right]^{\frac{1}{p}}
$$

8 $$ w_{{\text{V}}} + w_{{\text{S}}} = 1 $$

9 $$ p = 1 $$

(C) Comparison of inter-joint distances before and after optimum design

We compared the motion volume and obstructed value using three RF configurations with inter-joint distances on the Pareto frontier and one RF configuration with inter-joint distances prior to optimization. Specifically, we examined the following RF configurations:

I. An RF with a Pareto-optimal solution that minimizes the motion volume.
II. An RF with a Pareto-optimal solution that improves both the motion volume and obstructed value by the same ratio.
III. An RF with a Pareto-optimal solution that minimizes the obstructed value.
IV. An RF with $l_{a}$, $l_{b}$, and $l_{c}$ set to 3.5 mm, representing the RF configuration before optimization.

The inter-joint distances of RF I–IV for each participant are shown in Table 3.

**Table 3:** Parameters of robotic forceps I–IV

Participant ID	No	Parameters [mm]			Participant ID	No	Parameters [mm]		
		*l*_*a*_	*l*_*b*_	*l*_*c*_			*l*_*a*_	*l*_*b*_	*l*_*c*_
A	I_A_	2.0	2.0	2.0	C	I_C_	2.1	2.0	2.0
A	II_A_	2.0	3.6	3.0	C	II_C_	2.0	4.6	2.3
A	III_A_	2.3	3.4	3.2	C	III_C_	2.6	4.5	4.1
A	IV_A_	3.5	3.5	3.5	C	IV_C_	3.5	3.5	3.5
B	I_B_	2.1	2.0	2.0	D	I_D_	2.0	2.0	2.1
B	II_B_	2.0	3.0	2.0	D	II_D_	2.0	4.2	2.0
B	III_B_	2.1	3.0	2.0	D	III_D_	2.7	3.7	3.4
B	IV_B_	3.5	3.5	3.5	D	IV_D_	3.5	3.5	3.5

The participants were the same four pediatric surgeons described in Sect. "Experimental method"(A), and each task was conducted five times after the participants had become sufficiently familiar with it.

For each participant, five datasets were collected: Data I-5, Data II-5, Data III-5, and Data IV-5. Specifically, Data I-5 corresponded to the fifth trial of RF I, Data II-5 to the fifth trial of RF II, Data III-5 to the fifth trial of RF III, and Data IV-5 to the fifth trial of RF IV. Using these datasets, we conducted automatic simulations for each of the four RF configurations, and the motion volume and obstructed value were evaluated for each participant.

The Friedman test was used for statistical analysis. When a significant difference was found, multiple comparisons were conducted using the Holm correction. The significance level was set at p < 0.05. Statistical analysis was performed using R Version 4.4.2 (R Foundation for Statistical Computing, Vienna, Austria).

## Results

(A) Verification of the relationship between inter-joint distances and the motion volume and obstructed value

**Fig. 6:**
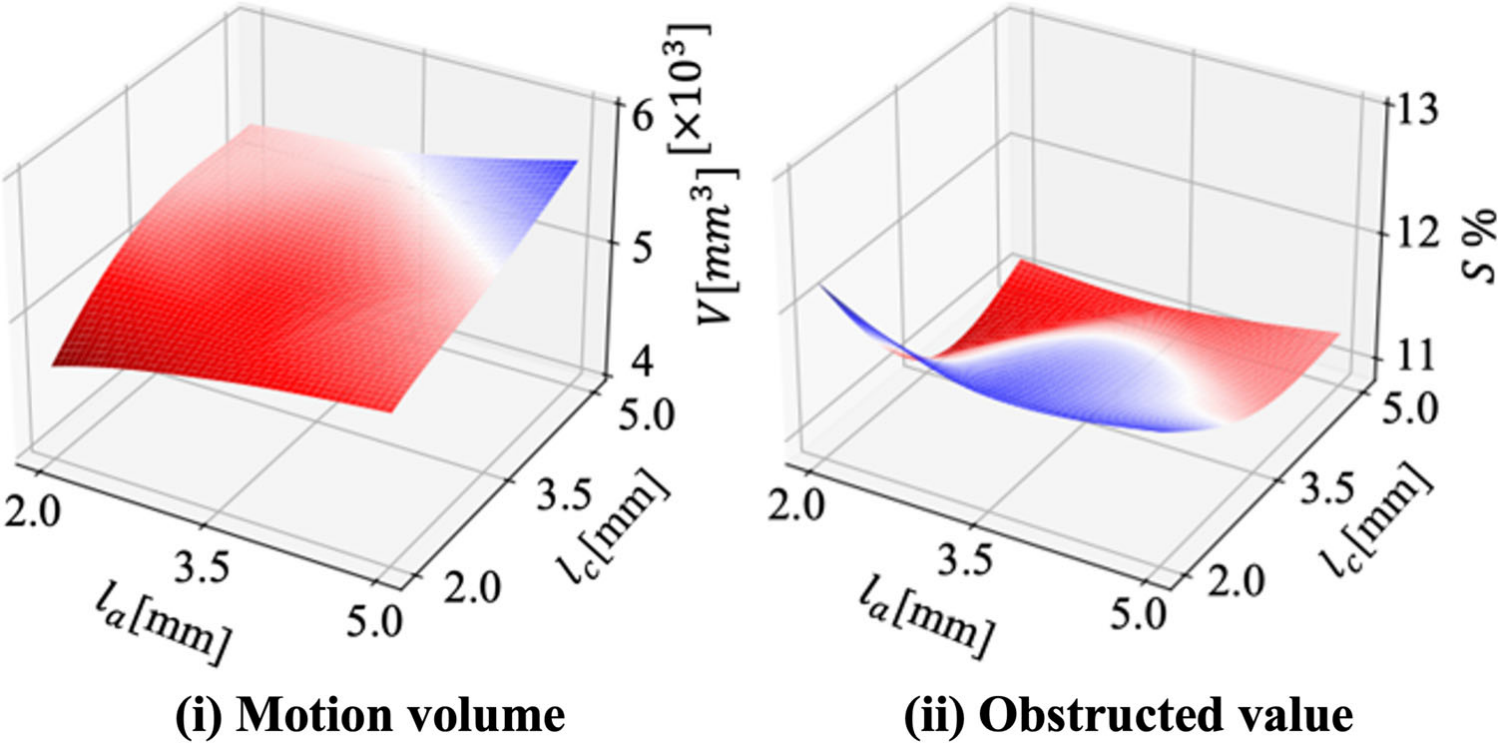
Relationships between inter-joint distances and assessment parameters (Participant A)

We confirmed the relationships between $l_{a}$, $l_{b}$, and the motion volume, between $l_{a}$, $l_{c}$, and the motion volume, and between $l_{b}$, $l_{c}$, and the motion volume. The relationship between $l_{a}$, $l_{c}$, and the motion volume for Participant A is shown in Fig. 6-(i) as part of the results. In Fig. 6-(i), $x$*-*, $y$-, and $z$-axes denote the values of $l_{a}$, $l_{c}$, and the motion volume $V$, respectively. From the results, we observed that the motion volume $V$ decreased as the values of $l_{a}$, $l_{b}$, and $l_{c}$, decreased (Fig. 6(i)). We confirmed the relationship between $l_{a}$, $l_{b}$, and the obstructed value, the relationship between $l_{a}$, $l_{c}$, and the obstructed value, and the relationship between $l_{b}$, $l_{c}$, and the obstructed value. The relationship between $l_{a}$, $l_{c}$, and the obstructed value of Participant A is shown in Fig. 6-(ii) as a part of the results. In Fig. 6(ii), $x$*-*, $y$*-*, and $z$*-*axes denote the values of $l_{a}$, $l_{c}$, and the obstructed value $S$. From the results, we observed that the obstructed value $S$ decreased with increasing values of $l_{b}$ and $l_{c}$ as shown in Fig. 6(ii). We also observed that the change in the obstructed value was affected by $l_{a}$ less than $l_{b}$ and $l_{c}$.

(B) Optimum design for inter-joint distances

From the results presented in Sect. "Results" (A), the approximate equations for the objective functions and design variables were calculated for each participant, as shown in Eqs. (10)–(17).

10 $$
 V_{{\text{A}}} = - 0.397 + 0.0829l_{a} + 0.0989l_{c} + 0.00875l_{b}^{2} + 0.0106l_{b} l_{c}
$$

11 $$
\begin{aligned}
S_{{\text{A}}} &= 2.93 - 0245l_{a} - 0.587l_{b} - 0.363l_{c} \\
&\quad + 0.0930l_{a} l_{b} + 0.0145l_{b} l_{c}^{2} \\
&\quad + 0.0107l_{b}^{2} l_{c} - 0.0181l_{a} l_{b} l_{c}
\end{aligned}
$$

12 $$
 V_{{\text{B}}} = - 0.286 + 0.0892l_{c} + 0.0154l_{a} l_{b} + 0.00975l_{b} l_{c} + 0.0114l_{c} l_{a}
$$

13 $$
\begin{aligned} S_{{\text{B}}} = 4.21 - 0.663l_{a} - 1.21l_{b} - 0.380l_{c} + 0.0427l_{a}^{2} + 0.129l_{b}^{2} + 0.172l_{a} l_{b} - 0.0163l_{a} l_{b}^{2} + 0.00853l_{b}^{2} l_{c} + 0.0626l_{c}^{2} l_{a} - 000939l_{a} l_{b} l_{c} \end{aligned}
$$

14 $$ V_{{\text{C}}} = - 0.362 + 0.106l_{a} + 0.0128l_{b}^{2} + 0.0204l_{c}^{2} $$

15 $$
\begin{aligned} S_{{\text{C}}} = 3.41 - 0.605l_{a} - 0.509l_{b} - 0.574l_{c} + 0.0276l_{a}^{2} + 0.0228l_{c}^{2} + 0.0934l_{b} l_{c} + 0.104l_{c} l_{a} + 0.0140l_{b} l_{c}^{2} - 00217l_{a} l_{b} l_{c} \end{aligned}
$$

16 $$ V_{{\text{D}}} = - 0.421 + 0.139l_{c} + 0.0155l_{a}^{2} + 0.0168l_{b}^{2} $$

17 $$
\begin{aligned} S_{{\text{D}}} = 3.25 - 0.282l_{a} - 0.267l_{b} - 1.05l_{c} + 0.0375l_{a}^{2} + 0.151l_{c}^{2} - 00143l_{b}^{2} l_{c} + 0.0166l_{b} l_{c}^{2} \end{aligned}
$$

We confirmed that the approximate equations had no accuracy problems because the predictor variables listed in Table 4 were greater than 0.5. We then performed the optimum design process and developed RF I–IV for each participant, as described in Sect. "Experimental method" (C) (Table 3).

**Table 4:** Predictor variables

	Predictor variables	
	Motion volume	Obstructed value
A	0.98	0.95
B	0.97	0.98
C	0.95	0.97
D	0.95	0.90

(C) Comparison of inter-joint distances before and after optimum design

**Fig. 7:**
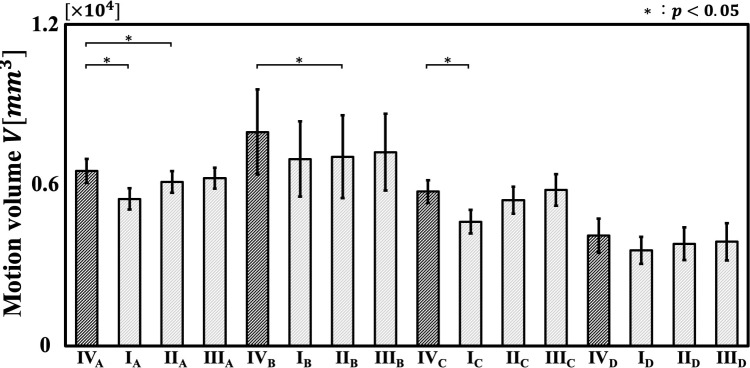
Motion volume of robotic forceps I–IV

The relationship between RF I–IV and motion volume is shown in Fig. 7, where the horizontal axis represents the different RF configurations for each participant, and the vertical axis represents motion volume. Compared to RF IV (before optimization), RF I exhibited a significantly smaller motion volume for Participants A and C, and RF II showed a significantly smaller motion volume for Participants A and B.

**Fig. 8:**
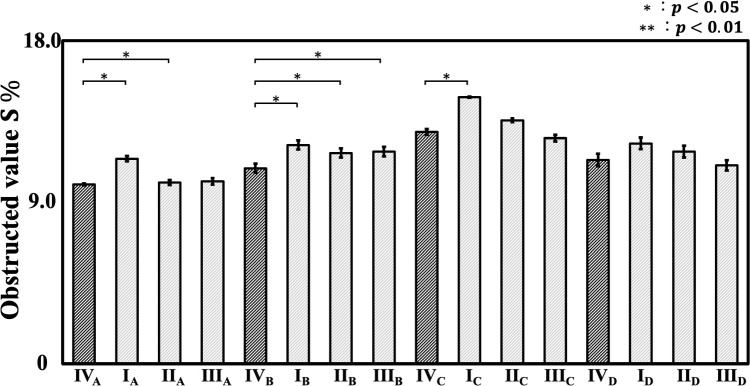
Obstructed value of robotic forceps I–IV

The relationship between RF I–IV and obstructed value is shown in Fig. 8, where the horizontal axis represents the different RF configurations for each participant, and the vertical axis represents obstructed value. Although no significant differences in obstructed values were observed for Participants A and D, RF II and III, which were expected to reduce obstruction, showed no significant improvement for Participant C. Additionally, for Participant B, the obstructed values of RF I, II, and III were significantly higher than those of RF IV.

## Discussion

### Verification of the relationship between inter-joint distances and the motion volume and the obstructed value

We observed that the motion volume decreased as the inter-joint distances is reduced in Fig. 7. This was due to the shortening of $l_{a}$, $l_{b} ,$ and $l_{c}$, which reduced the wrist’s radius of curvature during flexion. The decrease in motion volume was attributed to the reduced movement of the RF’s grasping part as the inter-joint distances shortened.

Conversely, the obstructed value decreased as $l_{b}$ and $l_{c}$ was increased. This was because the extension of these distances moved the RF further from the endoscope, reducing its size in the endoscopic view and subsequently lowering obstruction.

These findings highlight a trade-off between motion volume and the obstructed value for $l_{b}$ and $l_{c}$. This is attributed to the change in the wrist’s radius of curvature. This suggests that optimizing the inter-joint distances is crucial for minimizing the motion volume and obstructed value during vertical needle driving.

### Comparison of inter-joint distances before and after optimum design

**Fig. 9:**
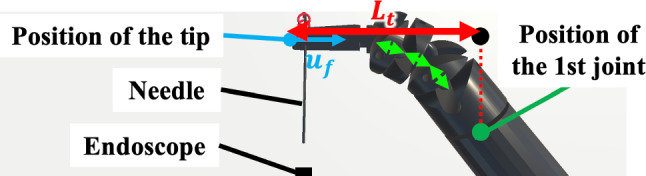
Distance between the position of the tip and that of the first joint

We observed improvements in motion volume by adjusting the inter-joint distances in RF I_A_, II_A_, II_B_, and I_C_. Specifically, in RF I_A_, II_B_, and I_C_, we confirmed that $L_{t}$, the distance between the tip and the first joint along the grasping direction ($u_{t}$), was shorter than in the RF before the optimum design (Fig. 9). This reduction was attributed to the shortening of $l_{a}$, $l_{b} ,$ and $l_{c}$, which decreased the wrist’s radius of curvature during flexion. Consequently, the motion volumes of RF I_A_, II_B_, and I_C_ decreased due to reduced movement in $u_{t}$. For RF II_A_, the reduction in motion volume was primarily due to the shortening of $l_{a}$ and $l_{c}$. However, $L_{t}$ in RF II_A_ was similar to that in the RF before the optimum design, indicating that additional analysis of the angular deflection and joint trajectory is required to determine the cause of this reduction. In Participant D, adjusting inter-joint distances did not lead to improvements in motion volume. A tendency toward decreased motion volume was observed in RF I_D_ and II_D_, but the differences were not statistically significant, likely due to the participant’s consistent operating tendencies. This task, involving passing a single needle through two rings, was simpler than actual clinical procedures. The lack of change in motion volume may be explained by the participant’s stable operation, developed through clinical experience.

**Fig. 10:**
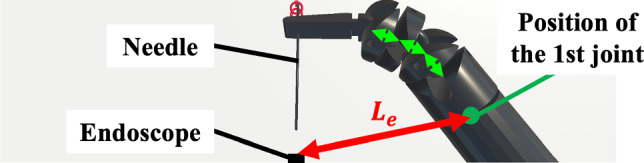
Distance between the position of the endoscope and that of the first joint

For all participants, adjusting inter-joint distances, including RF II and III, which were expected to improve obstruction, did not yield improvements in the obstructed value. In RF II_A_, III_A_, II_C_, III_C_, and III_D_, $L_{e}$, the distance between the endoscope and the first joint while grasping the needle (Fig. 10), was similar to that of the RF before the optimum design. This suggests that the obstructed value remained unchanged because the RF’s size in the endoscopic view was largely unaffected. The five-joint structure of the RF used in this study may have contributed to this stability. A previous study [23] indicated that five-joint RFs exhibited the smallest obstructed values among compared conditions during vertical needle driving. This implies that the impact of inter-joint distances on the obstructed value may be limited when the number of joints remains constant. Future studies should investigate the effect of inter-joint distances on motion volume and obstruction in RFs with different joint configurations.

In RF II_D_, $L_{e}$ was shorter than in the RF before the optimum design, suggesting that RF II_D_ may have moved closer to the endoscope. However, no significant difference in the obstructed value was found, likely due to the participants’ experience in clinical settings, where they consciously avoid obstructing their view while operating the RF. The obstructed value may be more sensitive to changes in task difficulty. Therefore, further investigation in an environment that closely mimics clinical conditions is necessary.

The obstructed values in RF II_B_ and III_B_ were worse than those in the RF before the optimum design. As observed in Sect. "Results"(A), the obstructed value increased when $l_{b}$ and $l_{c}$ decreased. It is possible that the expected improvement in obstruction was not achieved because $l_{b}$ and $l_{c}$ in the optimized RF were smaller than those in the pre-optimized RF. Further validation using other Pareto-optimal solutions is needed.

This study evaluated three Pareto-optimal solutions selected from the Pareto frontier; however, other solutions may exist that simultaneously improve both motion volume and obstruction. The validity of the optimization approach presented here is based on the specific task of vertical needle driving to the bile duct during choledochojejunostomy for CBD. Future studies should explore its application in other anastomotic procedures and ductal organ surgeries. Additionally, this study focused on inter-joint distances in a right-handed five-joint RF. Further investigations should include number of joint, alternative tip joint structures, left-handed RF configurations, and control method.

## Conclusion

Surgical robots enhance operating accuracy, but existing systems are often too large for pediatric patients. Developing surgical robots suitable for pediatric surgery requires designing structures that can operate efficiently in confined workspaces while supporting complex techniques specific to pediatric procedures. In this study, we focused on optimizing the inter-joint distances of the RF to enhance its adaptability to the narrow surgical workspace and the vertical needle driving specific to pediatric surgery. Our findings provide valuable insights into the trade-off between motion volume and obstruction during vertical needle driving. The inter-joint distances were adjusted using an optimum design, and we compared the RF before and after optimization to evaluate changes in motion volume and obstructed value. We observed improvements in motion volume for Participants A, B, and C, suggesting that adjusting inter-joint distances could promote adaptation to the narrow surgical workspace during vertical needle driving in choledochojejunostomy for CBD. However, the obstructed value did not improve for any participant, which was likely due to the five-joint RF structure used in this study. Therefore, further research is necessary to assess the impact of inter-joint distances on motion volume and obstructed value in RFs with different numbers of joints. The findings offer a foundation for future research on RF adaptability in constrained environments, particularly for choledochojejunostomy for CBD, advancing robotic surgery techniques and improving clinical outcomes in pediatric surgery.
